# Prevalence and factors associated with non-suicidal self-Injury and suicidality in children and adolescent attending Emergency department in Oman

**DOI:** 10.1192/j.eurpsy.2023.1561

**Published:** 2023-07-19

**Authors:** R. Alamri

**Affiliations:** 1 Oman, Muscat, Oman

## Abstract

**Introduction:**

Non-suicidal self-Injury (NSSI) and suicidality are common reasons for emergency presentations in child and adolescent psychiatry (1). NSSI is defined as intentional destruction of one’s body tissue without suicidal intent and has a prevalence rate in adolescents of approximately 30–40% in clinical samples (2). Suicide prevalence is around 24–33% (3).

There are many factors leading to suicidal or self-harming behavior. A prior history of self-injurious behavior is one of the strongest predictors of future suicidal behavior, both cross-sectionally and longitudinally (6) . Additionally, longitudinal studies have found that a previous suicide attempt increases the risk of a future suicide attempt threefold.

Suicide and NSSI have a significant impact on families and communities. Hence, considerable required clinical attention is warranted to develop preventive strategies.

**Objectives:**

The aim of this study is to investigate the prevalence of non-suicidal self-Injury (NSSI) and suicidality among children and adolescents presenting in emergency department of the tertiary psychiatric services and to study their demographic and clinical characteristics.

**Methods:**

This is a retrospective cross-sectional analytical study included all children and adolescent patients attended the emergency department at Sultan Qaboos University Hospital between June 2021 to March 2016. The data was collected using the hospital´s electronic database to retrieve the medical records of children and adolescents who visited the emergency department. Patients who were 18 years of the age or younger were included in the study. The included patients must had been evaluated by psychiatrist during their presentations in the emergency department.

**Results:**

During the 63 months of observation, 114 patients attended the emergency department and required psychiatric evaluation, 44.7%(n=51) of patients presented with NSSI and/or SA. The mean age was 15.7. 80.4%(n=41) were females while 19.6%(n=10) were males. 37.3% had a primary diagnosis of major depressive disorder (MDD) and 21.5% had comorbid medical illness. 44% of suicidal attempts were with drug overdose, mostly paracetamol overdose, while the most used method for NSSI was cutting the body with a razor, 57%.19 patients had a primary diagnosis of major depressive disorder, 17 patients had no clear diagnosis at presentation.22% of the patients had other medical comorbidities, 5 patients with epilepsy. 51% of the patients had positive family history of mental illness.

**Conclusions:**

Considering that NSSI and suicidality were found to be the main reasons for presenting to a child and adolescent emergency psychiatric service, it seems crucial for physicians at PEDs to provide proper crisis intervention and referral to mental health services when appropriate. Early identification of risk factors is highly recommended.

**Disclosure of Interest:**

None Declared

